# Formononetin Attenuates Renal Tubular Injury and Mitochondrial Damage in Diabetic Nephropathy Partly via Regulating Sirt1/PGC-1α Pathway

**DOI:** 10.3389/fphar.2022.901234

**Published:** 2022-05-12

**Authors:** Qunwei Huang, Hongbo Chen, Kai Yin, Yilan Shen, Kanghong Lin, Xieyi Guo, Xiang Zhang, Niansong Wang, Wenfeng Xin, Youhua Xu, Dingkun Gui

**Affiliations:** ^1^ Department of Nephrology, Shanghai Jiao Tong University Affiliated Sixth People’s Hospital, Shanghai, China; ^2^ Department of Nephrology, The First Affiliated Hospital of Zhejiang Chinese Medical University (Zhejiang Provincial Hospital of Traditional Chinese Medicine), Hangzhou, China; ^3^ Department of Cardiology, The Second Affiliated Hospital of Guilin Medical University, Guangxi Key Laboratory of Diabetic Systems Medicine, Guilin, China; ^4^ Guangxi Health Commission Key Laboratory of Glucose and Lipid Metabolism Disorders, The Second Affiliated Hospital of Guilin Medical University, Guilin, China; ^5^ Graduate School of Jiangxi University of Chinese Medicine, Nanchang, China; ^6^ College of Notoginseng Medicine and Pharmacy of Wenshan University, Wenshan, China; ^7^ Faculty of Chinese Medicine, State Key Laboratory of Quality Research in Chinese Medicine, Macau University of Science and Technology, Macao, China

**Keywords:** diabetic nephropathy, formononetin, tubular injury, mitochondrial dynamics, Sirt1/PGC-1α

## Abstract

Mitochondrial abnormality is one of the main factors of tubular injury in diabetic nephropathy (DN). Formononetin (FMN), a novel isoflavonoid isolated from *Astragalus* membranaceus, has diverse pharmacological activities. However, the beneficial effects of FMN on renal tubular impairment and mitochondrial dysfunction in DN have yet to be studied. In this study, we performed *in vivo* tests in Streptozotocin (STZ) -induced diabetic rats to explore the therapeutic effects of FMN on DN. We demonstrated that FMN could ameliorate albuminuria and renal histopathology. FMN attenuated renal tubular cells apoptosis, mitochondrial fragmentation and restored expression of mitochondrial dynamics-associated proteins, such as Drp1, Fis1 and Mfn2, as well as apoptosis-related proteins, such as Bax, Bcl-2 and cleaved-caspase-3. Moreover, FMN upregulated the protein expression of Sirt1 and PGC-1α in diabetic kidneys. *In vitro* studies further demonstrated that FMN could inhibit high glucose-induced apoptosis of HK-2 cells. FMN also reduced the production of mitochondrial superoxide and alleviated mitochondrial membrane potential (MMP) loss. Furthermore, FMN partially restored the protein expression of Drp1, Fis1 and Mfn2, Bax, Bcl-2, cleaved-caspase-3, Sirt1 and PGC-1α in HK-2 cells exposure to high glucose. In conclusion, FMN could attenuate renal tubular injury and mitochondrial damage in DN partly by regulating Sirt1/PGC-1α pathway.

## Introduction

Diabetes mellitus is one of the most common chronic diseases in the world and brings a heavy socioeconomic burden to the whole society. According to statistics, diabetes prevalence in people aged 20–79 years old was approximately 10.5 percent (536.6 million) in 2021 and will increase to 12.2 percent (783.2 million) in 2045 (Sun et al., 2022). Diabetic nephropathy (DN) is the main cause of end-stage renal disease (ESRD) (Cheng HT. et al., 2021). In China, with the increasing incidence and prevalence of DN, the proportion of diabetic-related chronic kidney disease outnumbered those with glomerulonephritis-related chronic kidney disease (Zhang et al., 2016). Glucose and blood pressure management are still the main therapeutic strategies according to KDIGO (2020) Clinical Practice Guideline (2020). However, it has been demonstrated that these strategies cannot fully stop or reverse the progression of DN (Samsu, 2021). Thus, there is still a great demand for new effective therapies for DN.

Renal tubular damage appears to have a key role in DN, according to growing evidence (Han et al., 2021; Yang et al., 2021; Li et al., 2022), and mitochondrial abnormalities largely contribute to this process (Xiang J. et al., 2022; Liu et al., 2022). Mitochondria is in a dynamic equilibrium of fission and fusion, which is called mitochondrial dynamics, and the dynamic balance is essential for maintaining normal mitochondrial structure, number, distribution, and function (Youle and van der Bliek, 2012; Chan, 2020). Drp1 and Fis1 play critical roles in regulating mitochondrial fission (McBride et al., 2006; Osellame et al., 2016) and mitochondrial fusion is mainly modulated by Mfn1, Mfn2 and OPA1 (Cipolat et al., 2006; Frezza et al., 2006; Suen et al., 2008). Excessive mitochondrial fission is associated with mitochondrial dysfunction and production of intracellular reactive oxygen species (ROS), which can cause damage to renal tubular cells (Zhan et al., 2015). These findings imply that the disturbance of normal mitochondrial dynamics may be linked to renal function decline and renal tubular injury. Thus, improving mitochondrial homeostasis may provide a novel strategy for the treatment of DN.

Mammalian silent information regulator two homolog-1 (Sirt1) is an NAD^+^-dependent protein deacetylase, which has been proved to have renoprotective effects (Morigi et al., 2018). Sirt1 has been reported to play beneficial roles in DN, such as anti-inflammatory and reducing oxidative stress (Sun et al., 2021; Qiu et al., 2022). Peroxisome proliferator receptor-γ coactivator 1 (PGC-1α) is a significant downstream target of Sirt1. It is crucial for the control of mitochondrial biogenesis and function (Lee et al., 2019). And there is evidence that Sirt1/PGC-1α axis is involved in mitochondrial dynamics (Cui et al., 2021).

Traditional Chinese medicine (TCM) has a long history of treating different kidney diseases in China. *Astragalus* membranaceus (huang qi) is a benefiting vital energy (Yiqi) herb in TCM that is widely used to treat DN. Our previous study found that Astragaloside II, an active ingredient of *Astragalus* membranaceus saponins, could protect podocyte from damage in STZ-induced diabetic rats (Su et al., 2021). Formononetin (FMN), one of the major isoflavonoid constituents isolated from *Astragalus* membranaceus, has been reported to have antioxidant activities (Fang et al., 2020). However, the protective effects of FMN on renal tubular injury in DN are yet to be studied. The purpose of this study was to investigate the beneficial effects of FMN on renal tubular injury and mitochondrial dysfunction in DN and to provide a novel treatment strategy for DN.

## Materials and Methods

### Drug Preparation

FMN (HPLC purity above 98%) was purchased from Shanghai Standard Technology Co. Ltd. (Shanghai, China). Losartan was obtained from Merck Sharp and Dohme Limited (Merck Sharp and Dohme, Australia). Streptozotocin (STZ) was purchased from Sigma-Aldrich Company (Sigma-Aldrich, United States). Both FMN and losartan were suspended in 0.5% methylcellulose solution for administration to rats. FMN was dissolved in DMSO to treat human proximal tubular epithelial cells (HK-2) exposed to high glucose. STZ was dissolved in citrate buffer (0.1 M, pH 4.5).

### Animal Studies

All animal studies were conducted in line with the National Institutes of Health (NIH) ’s Guide for the Care and Use of Laboratory Animals. The experimental protocols were approved by the Animal Ethics Committee of Shanghai Jiao Tong University Affiliated Sixth People’s Hospital. Eight-week-old male Sprague-Dawley rats weighing about 200–250 g were housed in a specific pathogen free (SPF) environment at a temperature of 18–23°C, relative humidity of 40–70%, and given standard clean diet and water. After 1 week of adaptive feeding, rats were given a single intraperitoneal injection of 55 mg/kg of STZ to induce diabetes. Rats having a blood glucose level above 16.7 mmol/L after 72 h of STZ injection were considered as diabetic rats. Rats were divided into four groups (n = 6/each group). Healthy normal rats without diabetes were considered as control (Con). Diabetic rats were randomly divided into three groups: 1) Diabetic rats induced by STZ, which received 0.5% methylcellulose solution in equal volume; 2) Diabetic rats treated with losartan (10 mg/kg/d) (Losartan); 3) Diabetic rats treated with FMN (20 mg/kg/d) (FMN). All treatments were intragastrically administrated to rats once daily for 8 weeks. Rats were placed in metabolic cages to collect 24 h urine. After 8 weeks of treatment, all rats were anesthetized with pentobarbital sodium, blood and kidneys were collected.

### Urine and Blood Biochemical Parameters

Urine was collected from metabolic cages, then centrifuged at 3,500 rpm for 15 min at 4C, and urinary albumin and urinary creatinine in the urine supernatants were measured with a fully automatic biochemical analyzer (Hitachi Model 7600-120E, Japan). The urine albumin/creatinine ratio (ACR) was then used to calculate urinary albumin excretion. Blood was collected from the abdominal aorta. The blood was left to stand for more than 30 min and centrifuged at 3,500 rpm for 15 min at 4°C, and the blood glucose (GLU), alanine aminotransferase (ALT) and serum creatinine (Scr) of the blood supernatants were measured with a fully automatic biochemical analyzer (Hitachi Model 7600-120E, Japan).

### Renal Histological Studies

The 4 μm sections of paraffin-embedded kidney tissues were stained with hematoxylin and eosin (HE) and periodic acid-Schiff (PAS). The protocols were previously described (Su et al., 2021). After drying at 65°C for 30 min, sections were deparaffinized in dimethylbenzene twice for 10 min each time. Then sections were rehydrated through 100% ethanol (I), 100% ethanol (II), 95% ethanol, 90% ethanol, 80% ethanol, and deionized water, 10 min for each step. Subsequently, sections were stained with HE and PAS solutions. 20 images (×200 magnification, bars = 50 µm) were collected randomly for quantification by two blinded researchers. The percentage of mesangial matrix occupying each glomerulus was calculated. The tubular injury score was graded and scored from 0 to 3 as follows: 0, no lesion; 1, <25% of tubules injured; 2, 25–50% of tubules injured; 3, >50% of tubules injured (Cheng D. et al., 2021).

### Transmission Electron Microscopy Studies

Foot processes (FP) of podocytes and the mitochondria in tubular cells of kidneys were observed by transmission electron microscopy (TEM). The protocol was described previously (Xie et al., 2020). Briefly, the renal cortex was fixed with 2% glutaraldehyde, stained with uranyl acetate and lead citrate. The number of foot processes (FP) was counted as previously described (Zhai et al., 2019).

### Immunohistochemistry

Immunohistochemistry was conducted on 4 μm paraffin-embedded renal tissues. Antigens were extracted by boiling in citrate buffer after deparaffinization and rehydration. Sections were blocked for 15 min with 0.3% H_2_O_2_ and 1 h with 5% BSA. Primary antibodies of Drp1 (1:200, Abcam, United States), Mfn2 (1:250, CST, USA), Fis1 (1:200, Abcam, United States), Sirt1 (1: 200, Abcam, USA), PGC-1α (1:200, ABclonal, China) were incubated overnight at 4°C. Then the sections were incubated with the secondary antibodies (Dako, United States) for 1 h at 37°C. Finally, the sections were counterstained with diaminobenzidine and hematoxylin. A light microscope (Leica, Germany) was then used to collect photomicrographs. And all photomicrographs were analyzed by ImageJ software.

### Immunofluorescence Staining and TUNEL Assay

Terminal deoxynucleotidyl transferase-mediated dUTP-biotin nick-end labeling (TUNEL) assay was utilized to detect the apoptosis of cells in kidneys. It was conducted on 4 μm frozen sections of kidney tissues using an Apoptosis Detection Kit (Merck, United States). Briefly, cells were fixed with 4% paraformaldehyde and washed twice with PBS for 10 min each. The sections were incubated with PBS containing 0.5% Triton X-100 at room temperature for 5 min. Then washed the sections twice with PBS and added the TUNEL detection solution. Nuclei were counterstained with DAPI. Images were collected by a fluorescence microscope (Leica, Germany).

### Cell Culture

HK-2 cells were cultured in DMEM medium containing 10% fetal bovine serum at 37°C, 5% CO_2_. Cells were trypsinized and passaged when they grew to 70–80% confluency. Cells in logarithmic phase were divided into five groups: 1) Con, cells cultured in 5.6 mM glucose; 2) HM, cells cultured in 5.6 mM glucose +24.5 mM mannitol; 3) HG, cells cultured in 30 mM glucose; 4) FMN-L, cells cultured in 30 mM glucose +10μM FMN; 5) FMN-H, cells cultured in 30 mM glucose +20 μM FMN. Each group was treated for 48 h.

### Flow Cytometry

The apoptosis of HK-2 cells was measured using the Annexin V-FITC/PI Apoptosis Detection Kit (BD, United States). Cells were trypsinized and resuspended in PBS after centrifugation. After centrifugation again, cells were resuspended in binding buffer. Cell suspensions were examined by a flow cytometer (Beckman Coulter, United States) after being incubated with Annexin V-FITC and PI in the dark.

### Living Cell Imaging

The production of mitochondrial superoxide was measured using MitoSOX Red mitochondrial superoxide indicator (Invitrogen, USA, 510–580 nm). It was dissolved in DMSO, then diluted 1:1,000 with cell culture medium, and incubated with cells for about 20 min. Mitochondrial membrane potential (MMP) levels were detected by a JC-1 detection kit (Beyotime Tech, China). First, we prepared the work solution according to the instructions. Then cells were incubated with the working solution and cell culture medium for about 20 min. JC-1 can transition between J-monomers and J-aggregates depending on the mitochondrial membrane potential level. J-monomers can produce green fluorescence, and the J-aggregates can produce red fluorescence. The membrane potential was expressed by the ratio of their fluorescence. The nuclei of cells were counterstained with Hoechst 33,342 (Beyotime Tech, China). A fluorescence microscope (Leica, Germany) was used to record images and the images were analyzed by ImageJ software.

### Western Blot

RIPA lysis buffer (Beyotime Tech, China) containing phosphatase and protease inhibitors was used to extract proteins from kidney tissues and cells. The tissue lysates were mixed with 5 × SDS buffer and denatured at 100 °C for 10 min. The samples were separated by SDS-polyacrylamide gel electrophoresis (SDS-page) and transferred to polyvinylidene fluoride (PVDF) membranes. The membranes were then blocked with 5% nonfat milk in TBST for 1 h at room temperature. Primary antibodies of Bcl-2 (1:1,000, Abcam, United States), Bax (1:1,000, CST, United States), cleaved-caspase-3 (1:500, CST, United States), Drp1 (1:1,000, Abcam, United States), Mfn2 (1:1,000, CST, United States), Fis1 (1:500, Abcam, United States), Sirt1 (1:1,000, Abcam, United States), PGC-1α (1:1,000, ABclonal, China), β-actin (1:5,000, Abcam, United States) were incubated with membranes overnight at 4°C. After washing with TBST for 5–10 min, the membranes were incubated at room temperature for 1 h with HRP combined secondary antibody (Abcam, United States) and then washed with TBST for 10 min for 3 times. Finally, the proteins bands were visualized with ECL and the band intensity was measured with ImageJ software.

### Statistical Analysis

GraphPad Prism eight was utilized to analyze the data (Markovics et al., 2020), and all data were expressed as means ± standard deviation (SD). One-way analysis of variance (ANOVA) was applied for differences among multiple groups. Statistical significance was defined as a *p* value < 0.05.

## Results

### Effects of FMN on Physical and Biochemical Characteristics in Diabetic Rats

**FIGURE 1:**
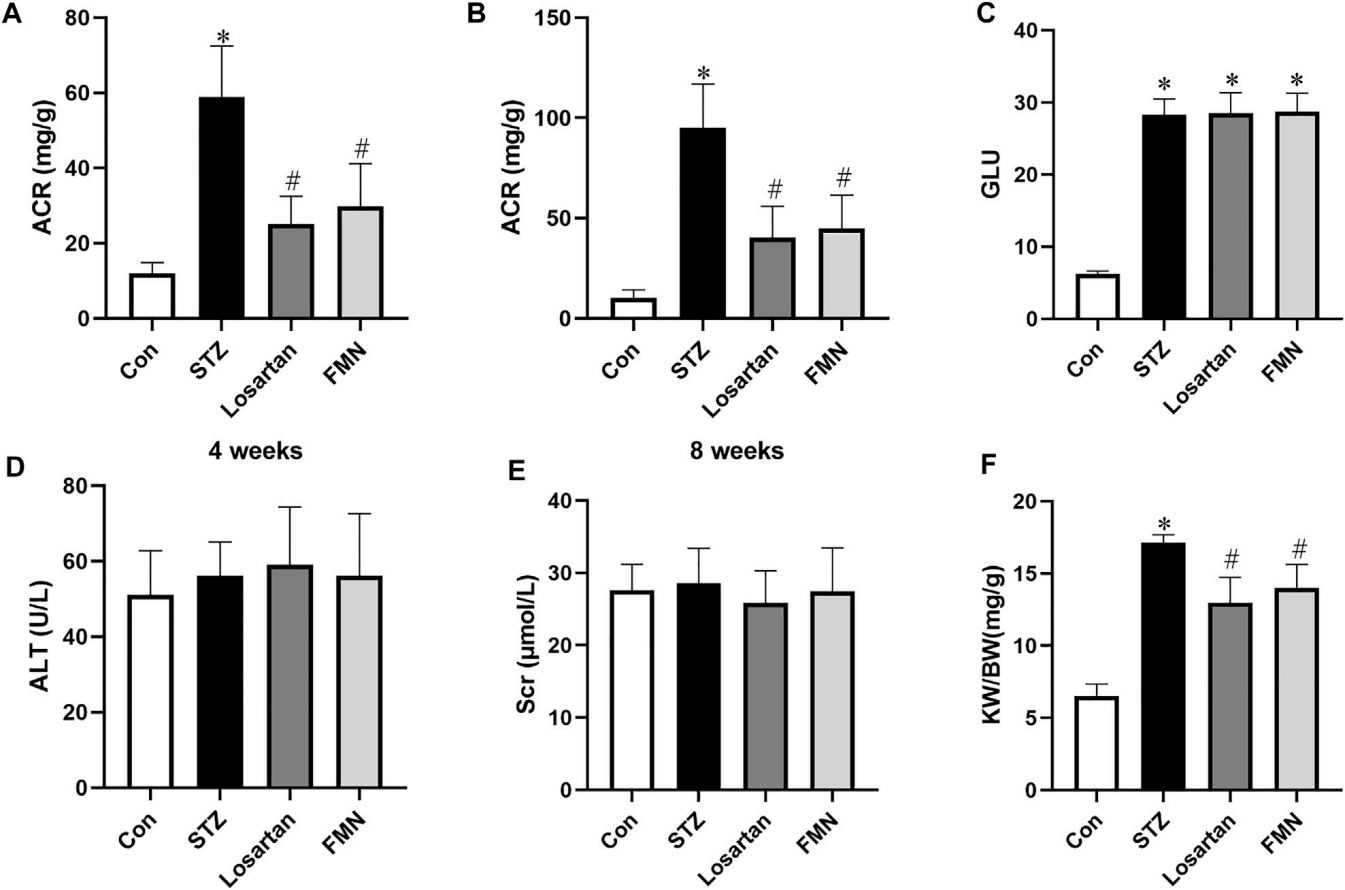
Effects of FMN on physical and biochemical characteristics in diabetic rats. **(A)** ACR levels after 4 weeks of treatment. **(B)** ACR levels after 8 weeks of treatment. Blood glucose (GLU) **(C)**, alanine aminotransferase (ALT) **(D)**, serum creatinine (Scr) **(E)** and renal index (KW/BW) **(F)** levels after 8 weeks of treatment. Results were expressed as mean ± SD (*n* = 6). *p < 0.05 vs. normal control rats. #*p* < 0.05 vs. STZ-induced diabetic rats.

Compared to normal control rats, the ACR level in diabetic rats was considerably higher. However, after 4 and 8 weeks of treatment, FMN or losartan significantly decreased ACR level in diabetic rats. (Figures 1A,B). There was no significant difference in blood glucose level in the FMN or losartan treatment group when compared with the diabetic model group (Figure 1C). And there was no significant difference in the levels of alanine aminotransferase (ALT) or serum creatinine (Scr) among the groups, suggesting that FMN has no apparent toxicity to liver or kidney (Figures 1D,E). In addition, the renal index (kidney weight per body weight ratio, KW/BW) was significantly increased in diabetic rats. After 8 weeks of treatment, FMN or losartan reduced the renal index in diabetic rats (Figure 1F).

### Effects of FMN on Renal Morphological Changes in Diabetic Rats

**FIGURE 2:**
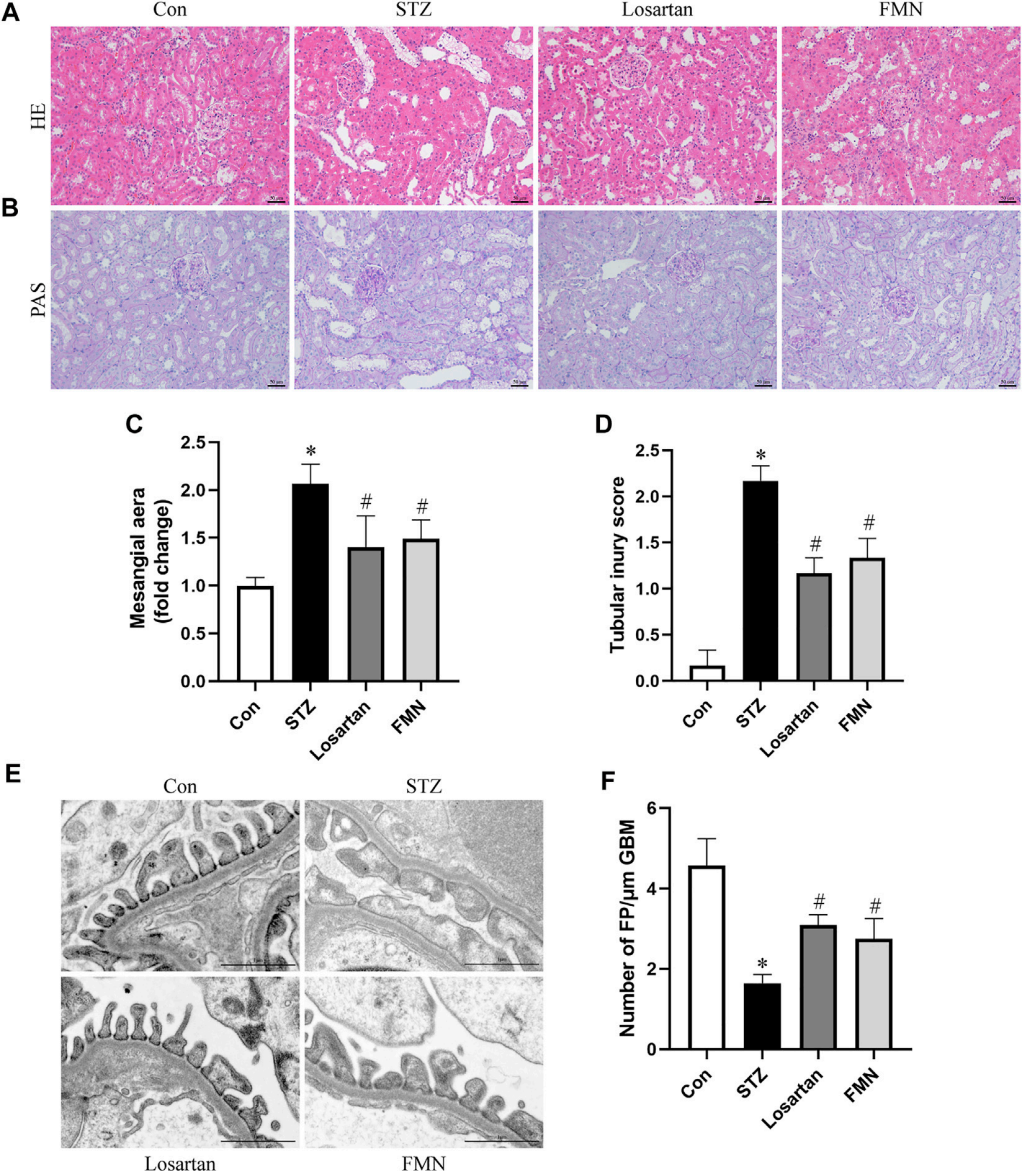
FMN improved renal histopathology and podocyte morphological abnormalities in diabetic rats. **(A,B)** Renal histology evaluations were performed with HE and PAS staining (original magnification × 200, bars = 50 µm). **(C,D)** Semiquantitative analyses of mesangial area changes and tubular injury score. **(E,F)** Ultrastructure images of podocytes collected by transmission electron microscopy (TEM) (original magnification × 8,000, bars = 1 µm) and semiquantitative analysis of podocyte FP density. Results were expressed as mean ± SD. *p < 0.05 vs. normal control rats. #*p* < 0.05 vs. STZ-induced diabetic rats.

Diabetic rats had notable mesangial matrix deposition, renal tubular dilatation and renal tubular vacuolar degeneration. These histopathological changes were detected by HE and PAS staining. Our results found that FMN or losartan significantly improved these pathological changes (Figures 2A–D). Furthermore, apparent podocyte loss, FP fusion, and effacement were observed by TEM in diabetic rats. These changes were partially reversed by FMN or losartan (Figures 2E,F). The above results indicated that FMN attenuated renal morphological abnormalities in diabetic rats.

### Effects of FMN on Renal Tubular Cells Apoptosis in the Kidneys of Diabetic Rats

**FIGURE 3:**
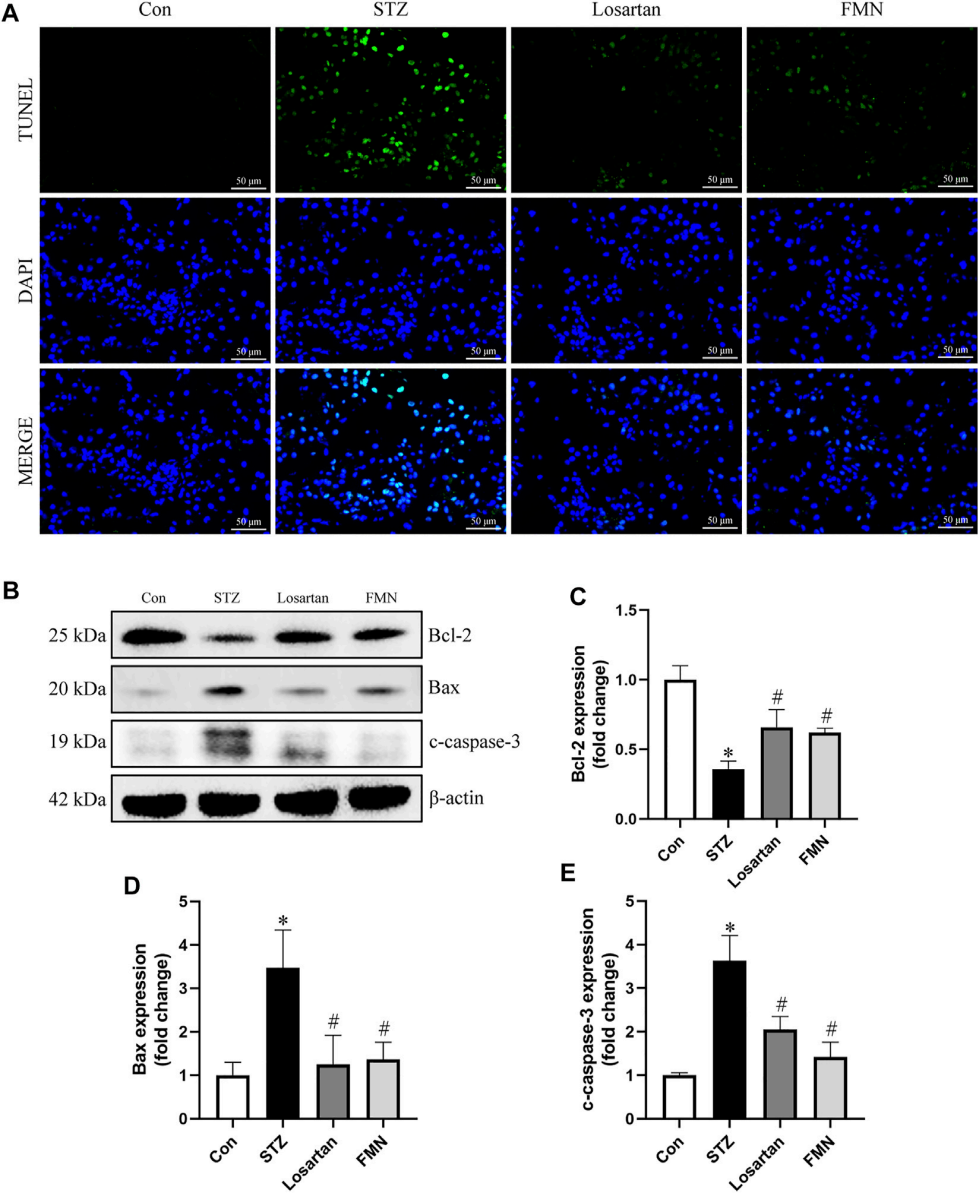
FMN attenuated renal tubular cells apoptosis in diabetic rats. **(A)** Representative double immunofluorescence labeling, including the TUNEL assay and DAPI on frozen kidney sections **(B–E)** Representative images of Bcl-2, Bax and c-caspase-3 protein expression and semiquantitative analyses in different groups. Results were expressed as mean ± SD. *p < 0.05 vs. normal control rats. #*p* < 0.05 vs. STZ-induced diabetic rats.

As shown in Figure 3, TUNEL-positive cells were found in greater numbers in the tubular compartment in the kidneys of diabetic rats by TUNEL assay. However, FMN or losartan could significantly attenuate the renal tubular cells apoptosis in STZ-induced diabetic rats (Figure 3A). We also detected the levels of apoptosis-related proteins in the kidneys of diabetic rats by western blot. Bcl-2, which was considered as an antiapoptotic protein, was significantly reduced in diabetic rats. In addition, proapoptotic proteins like Bax and cleaved-caspase-3 (c-caspase-3) were upregulated in diabetic kidneys. These abnormalities were partially reversed by FMN treatment (Figures 3B–E).

### Effects of FMN on Mitochondrial Morphology Changes and Dynamics-Associated Proteins Expression in Renal Tubular Cells

**FIGURE 4:**
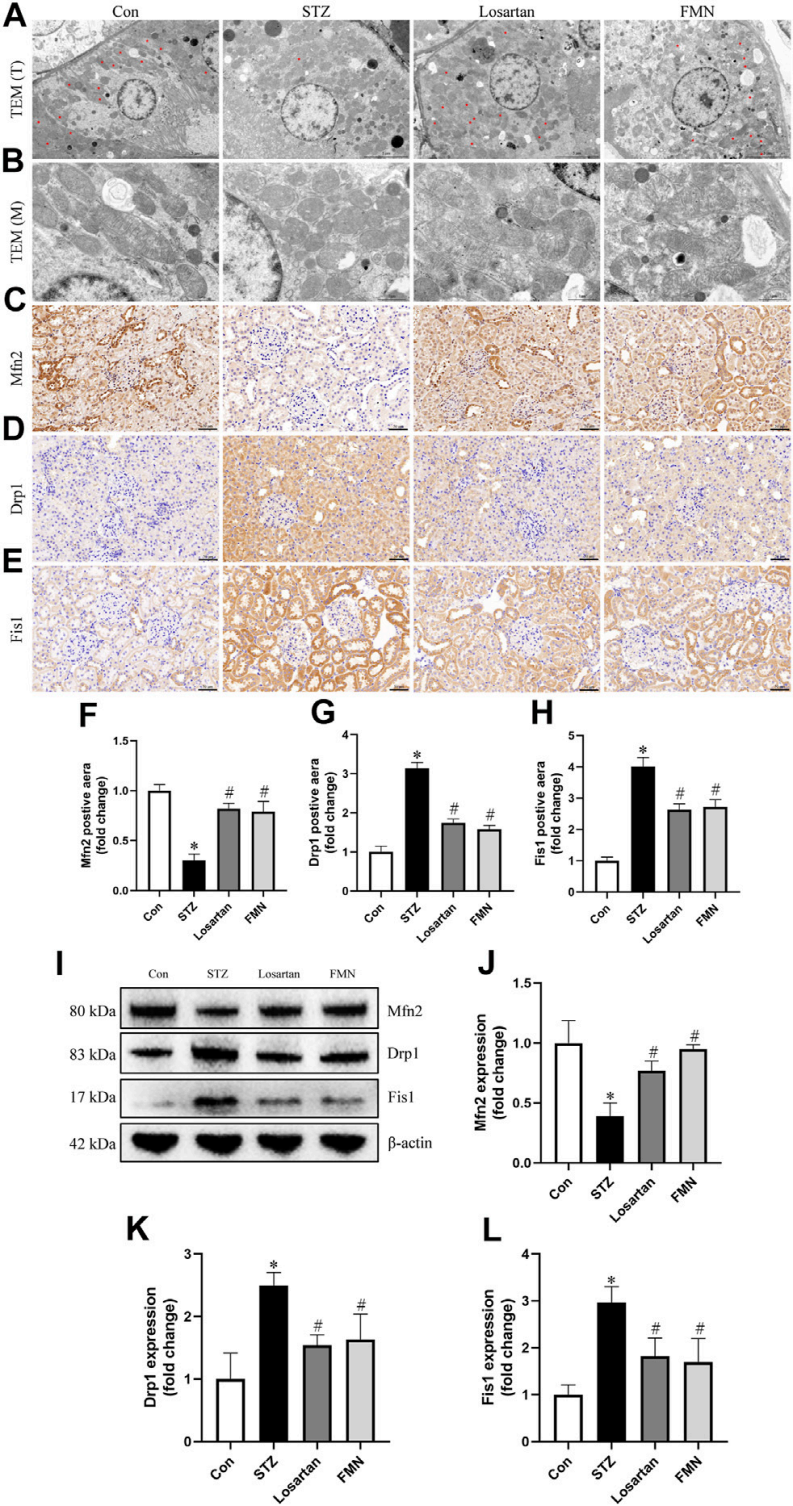
FMN ameliorated the mitochondrial morphology abnormalities in renal tubular cells and restored the expression of dynamics-associated proteins in diabetic rats. **(A)** Mitochondria morphology alterations in renal tubular cells observed by TEM (original magnification × 2,500, bars = 5 µm). T, tubules. Red asterisks, elongated cylindrical mitochondria. **(B)** High-magnification TEM micrographs of mitochondrial ultrastructure (original magnification × 8,000, bars = 1 µm). M, mitochondria. **(C–E)** Representative photographs of immunohistochemistry staining of Drp1, Fis1 and Mfn2 (original magnification ×200, bars = 50 µm). And semiquantitative analyses of immunohistochemistry of Mfn2 **(F)**, Drp1 **(G)** and Fis1 **(H)**. **(I–L)** Representative Western blot images of Mfn2, Drp1 and Fis1 protein expression and semiquantitative analyses. Results were showed as mean ± SD. *p < 0.05 vs. normal control rats. #*p* < 0.05 vs. STZ-induced diabetic rats.

Changes in mitochondrial morphology and dynamics-associated proteins were detected by using TEM, immunohistochemical labeling and western blot to further investigate the beneficial effects of FMN on mitochondrial homeostasis. As shown in Figures 4A,B, the majority of mitochondria in renal tubular cells were rod-shaped or spherical in diabetic rats, with partially disintegrating cristae. After 8 weeks treatment of FMN or losartan, these changes were partly improved.

As Drp1 and Fis1 are critical for mitochondrial fission, and Mfn2 regulates mitochondrial fusion, we thus investigated the protein expression of Drp1, Fis1 and Mfn2. When compared to normal control rats, the protein expression of Drp1 and Fis1 was significantly increased, while the protein expression of Mfn2 was decreased in kidneys from diabetic rats. However, FMN or losartan treatment decreased Drp1 and Fis1 expression while increased Mfn2 expression in diabetic kidneys (Figures 4C–L). These results indicated that FMN attenuated mitochondrial morphology and dynamics abnormalities in the kidneys of STZ-induced diabetic rats.

### Effects of FMN on Regulating Sirt1/PGC-1α Signaling Pathway in Diabetic Rats

**FIGURE 5:**
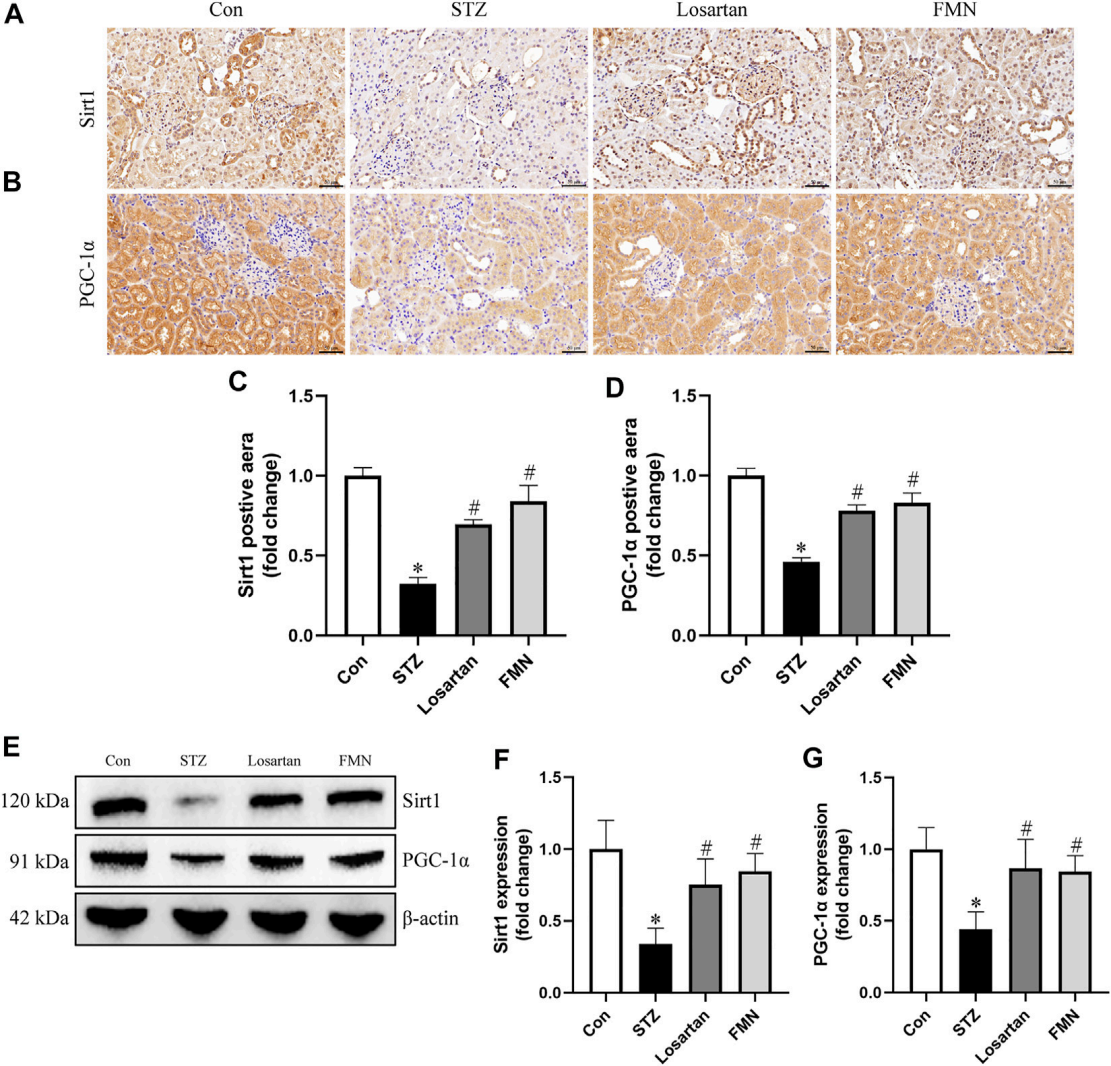
FMN restored the Sirt1 and PGC-1α expression in diabetic rats. **(A–D)** Representative photographs and semiquantitative analyses of immunohistochemistry staining of Sirt1 and PGC-1α (original magnification ×200, bars = 50 µm). **(E–G)** Representative Western blot images of Sirt1 and PGC-1α protein expression and semiquantitative analyses in different groups. Results were expressed as mean ± SD. *p < 0.05 vs. normal control rats. #*p* < 0.05 vs. STZ-induced diabetic rats.

The expression of Sirt1 and PGC-1α related to mitochondrial dynamics was investigated by immunohistochemistry staining and western blot. The protein expression of Sirt1 and PGC-1α was significantly reduced in the kidneys of diabetic rats. However, FMN or losartan treatment restored protein expression of Sirt1 and PGC-1α in diabetic kidneys (Figures 5A–G). These data indicated that FMN might exert the renoprotective effects partly by regulating Sirt1/PGC-1α axis.

### Effects of FMN on Inhibiting Apoptosis of HK-2 Cells Subjected to HG Exposure

**FIGURE 6:**
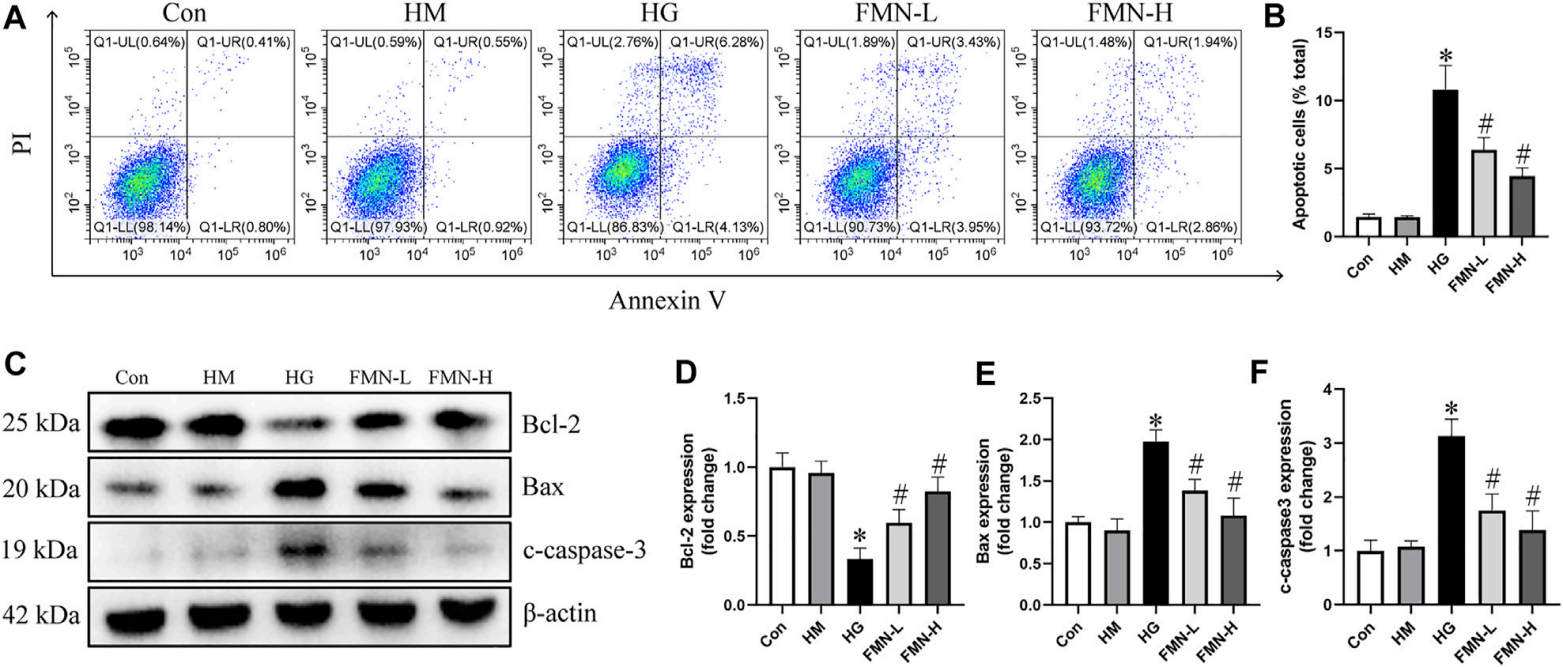
FMN reduced the apoptosis of HK-2 cells under HG exposure. **(A,B)** Representative images of flow cytometry and quantitative analysis of apoptosis rate in HK-2 cells. *n* = 3 **(C–F)** Representative images of Bcl-2, Bax and c-caspase-3 protein expression and semiquantitative analyses in different groups. Results were expressed as mean ± SD. *p < 0.05 vs. control group. #*p* < 0.05 vs. HG group.

The apoptosis rate of HK-2 cells was significantly increased in HG group after being treated with high glucose for 48 h. However, both 10 μM FMN and 20 μM FMN could significantly reduce HK-2 cells apoptosis (Figures 6A,B). There was no significant difference between hypertonic group and normal glucose control group. Additionally, we also investigated the levels of apoptosis-related proteins in HK-2 cells. High glucose treatment could significantly decrease the expression of Bcl-2 and increase the expression of Bax and c-caspase-3 in HK-2 cells. However, FMN partially restored these changes (Figures 6C–F). These results showed that FMN could protect HK-2 cells from apoptosis under high glucose condition.

### Effects of FMN on Mitochondrial Superoxide Production and Mitochondrial Membrane Potential in HK-2 Cells Subjected to HG Exposure

**FIGURE 7:**
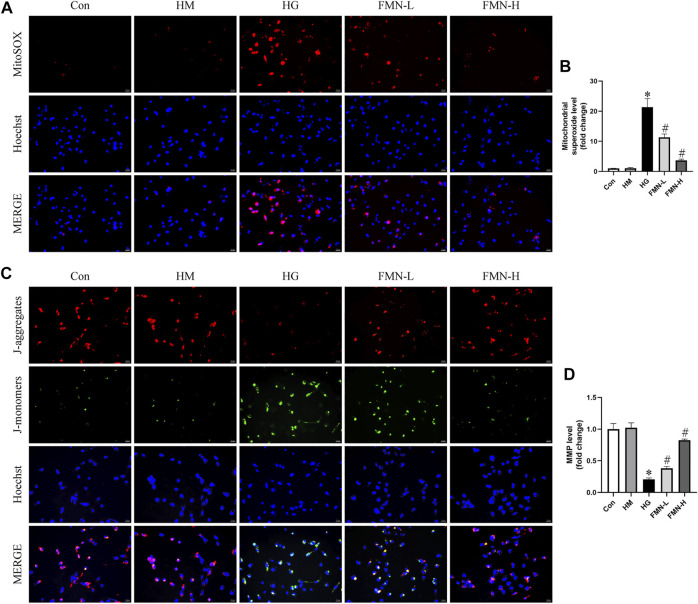
FMN reduced mitochondrial superoxide overproduction and restored MMP levels. **(A,B)** Representative images and semiquantitative analysis of mitochondrial superoxide production in HK-2 cells (original magnification ×200, bars = 50 µm). **(C,D)** Representative images and semiquantitative analysis of MMP levels in HK-2 cells (original magnification ×200, bars = 50 µm). Results were expressed as mean ± SD. *p < 0.05 vs. control group. #*p* < 0.05 vs. HG group.

Evidence indicates mitochondrial membrane depolarization, overproduction of ROS, and activation of mitochondrial cell death pathway occur in cells under stress (Zhan et al., 2015). To evaluate mitochondrial superoxide and MMP levels in HK-2 cells, we employed MitoSOX Red mitochondrial superoxide indicator and JC-1 staining. Normal levels of mitochondrial ROS are involved in maintaining cellular homeostasis, while excess mitochondrial ROS production can lead to cellular damage (Coughlan and Sharma, 2016). It seems HG caused mitochondrial superoxide overproduction in HK-2 cells. And this change was attenuated after FMN administration (Figures 7A,B). When intracellular mitochondrial membrane potential is high, JC-1 forms J-aggregates in the mitochondrial matrix, which can create red fluorescence. On the contrary, when the potential is low, since it cannot aggregate, JC-1 is a monomer which can emit green fluorescence. As shown in Figures 7C,D, HG could cause loss of MMP in HK-2 cells, and FMN reversed the mitochondrial membrane depolarization. The above results suggested FMN could restore the mitochondrial function in HK-2 cells exposed to HG.

### Effects of FMN on Mitochondrial Dynamics-Associated Proteins and Sirt1/PGC-1α Axis in HK-2 Cells Subjected to HG Exposure

**FIGURE 8:**
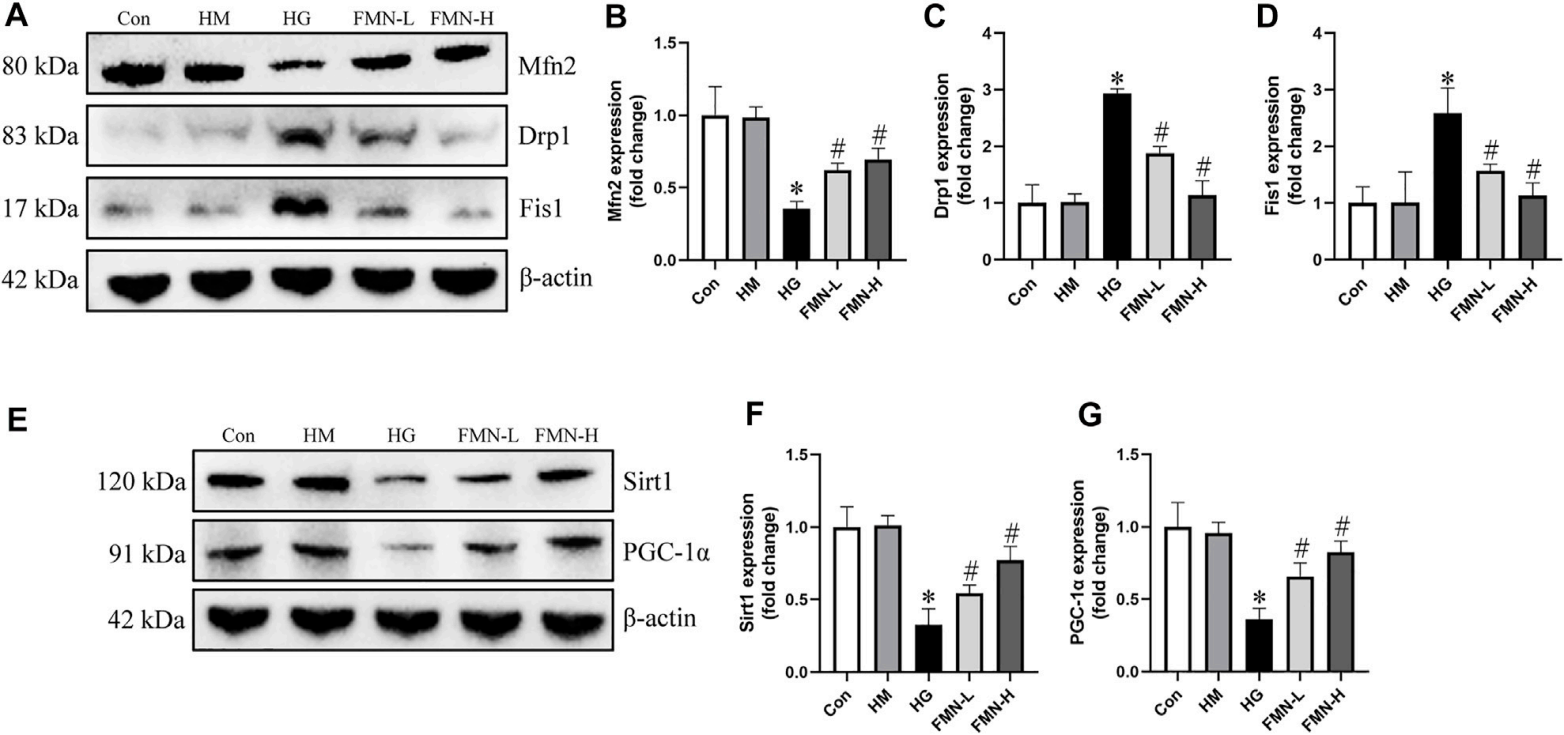
FMN restored the expression of mitochondrial dynamics-associated proteins and Sirt1/PGC-1α. **(A–D)** Representative Western blot images of Mfn2, Drp1 and Fis1 protein expression and semiquantitative analyses in different groups. **(E–G)** Western blot analyses and semiquantitative analyses of expression of Sirt1 and PGC-1α. Results were expressed as mean ± SD. *p < 0.05 vs. control group. #*p* < 0.05 vs. HG group.

We next investigated the effects of FMN on expression of mitochondrial dynamics-associated proteins. We found that the protein expression of Drp1 and Fis1 was upregulated, while Mfn2 was downregulated in HK-2 cells exposed to HG detected by western blot analysis. However, the expression of these proteins was restored by FMN treatment (Figures 8A–D). The expression of Sirt1 and PGC-1α was also detected in HK-2 cells. Western blot results showed HG significantly reduced the expression of Sirt1 and PGC-1α, while these abnormalities were partially restored by FMN treatment (Figures 8E–G). Therefore, these results demonstrated that FMN partly blocked HG-mediated mitochondrial dynamics and function abnormalities in tubular cells by regulating Sirt1/PGC-1α pathway.

## Discussion

FMN possesses a diverse set of pharmacological effects, such as anti-oxidative stress, anti-inflammatory, regulating blood glucose and lipid profile (Li et al., 2014; Oza and Kulkarni, 2018; Aladaileh et al., 2019; Xiang K. et al., 2022). However, the beneficial effects of FMN on renal tubular injury and mitochondrial damage in DN have yet to be investigated. This study demonstrated that FMN, a novel isoflavonoid constituent from *Astragalus* membranaceus, partially inhibited apoptosis and mitochondrial dysfunction of renal tubular cells in DN partly through the regulation of Sirt1/PGC-1α pathway. FMN dose-dependently inhibited high glucose (HG)-induced HK-2 cells apoptosis *in vitro*. *In vivo* studies further showed that the treatment with FMN significantly ameliorated albuminuria, renal histopathology, renal tubular cells apoptosis and mitochondrial damage in STZ-induced diabetic rats. Moreover, glucose-induced apoptosis of renal tubular cells was associated with upregulation of Bax expression and downregulation of Bcl-2 expression, as well as caspase-3 activation, and these abnormalities were partially restored by FMN *in vivo* and *in vitro*. Furthermore, FMN also improved the expression of mitochondrial dynamics-associated proteins, such as Drp1, Fis1 and Mfn2. Finally, FMN increased the protein expression of Sirt1 and PGC-1α *in vitro* and *in vivo*. Taken together, FMN partially ameliorated renal tubular injury and mitochondrial damage in DN partly through the regulation of Sirt1/PGC-1α pathway. These findings might contribute to the development of a novel therapeutic strategy for the treatment of DN.

Firstly, we investigated the effects of FMN on renal function and morphological changes in diabetic rats. Several studies explored the renal protective effects of FMN in type 2 diabetes models (Oza and Kulkarni, 2019; Lv et al., 2020; Zhuang et al., 2020). Our study aimed to investigate the protective effects of FMN on renal tubular impairment and mitochondrial dysfunction in STZ-induced type 1 diabetic rats. Losartan was used as a positive control as in our previous study (Su et al., 2021). FMN significantly reduced the albuminuria at 4 and 8 weeks after STZ injection. FMN also improved renal histopathology in STZ-induced diabetic rats. Furthermore, FMN ameliorated podocyte foot process effacement in diabetic rats. These results showed that FMN delayed the progression of DN in diabetic rats by improving renal function and structure abnormalities.

Recent studies have revealed that tubular injury plays a significant role in the development of DN, which is associated with renal functional impairment (Xiao et al., 2014; Xiao et al., 2017; Ma et al., 2018). Studies showed that tubular damage markers appear prior to the onset of microalbuminuria (Bonventre, 2012; de Carvalho et al., 2016), reflecting that tubular injury leads to primary renal injury. Another study found that the reabsorption of albumin in the renal proximal tubular cells in STZ-induced diabetic rats was significantly lower than that in the control group, but no changes in eGFR occurred in diabetic rats at this time, further confirming that the tubular injury occurs early in the DN (Tojo et al., 2001). The lesions of renal tubules even could cause glomerular damage (Gilbert, 2017).

Clinical study in patients with type 1 diabetes further showed the early involvement of the tubules in albuminuria (Gibb et al., 1989). The researchers suggested that albuminuria might origin from renal tubules in DN (Comper et al., 2008). Collectively, the causative factor of renal tubular injury in DN is supported by diabetic patients and experimental models and the renal tubules are heavily involved in the pathogenesis of DN. Thus, our study focused on the protective effects of FMN on renal tubule injury.

In this study, we detected the renal tubular cells apoptosis and the levels of apoptosis-related proteins *in vitro* and *in vivo*. FMN could significantly attenuated renal tubular cells apoptosis and restored the protein levels of Bcl-2, Bax and cleaved-caspase-3. The above results demonstrated that FMN attenuated renal tubular cells apoptosis in DN.

We next investigated the mechanisms underlying the action of FMN on renal tubular cells apoptosis in diabetic rats. Mitochondrial dysfunction is a major contributor to renal tubular injury (Zhan et al., 2015). Renal tubular cells have abundant mitochondria and their function relies on oxidative phosphorylation. The mitochondria maintain cell homeostasis and fulfill the high metabolic energy needs of renal tubular cells. Therefore, tubules are sensitive to mitochondrial dysfunction (Higgins and Coughlan, 2014). Accumulating evidence indicates that excessive mitochondrial fission leads to mitochondrial fragmentation, membrane depolarization and production of mitochondrial ROS under stress (Xiao et al., 2017; Zhang et al., 2020). Normal levels of mitochondrial ROS are involved in maintaining cellular homeostasis and normal intracellular signal transduction, while excess mitochondrial ROS production can lead to cellular damage (Coughlan and Sharma, 2016). It seems HG caused mitochondrial superoxide overproduction in HK-2 cells. And FMN could reverse this change. However, the simultaneous spatiotemporal measurements of ROS generation, compartmentalization, and the relation between ROS and cellular homeostasis need to be further investigated to elucidate the role of ROS in DN. We also found that FMN could significantly alleviate mitochondrial membrane potential loss in HK-2 cells subjected to high glucose exposure. FMN also reduced mitochondrial fragmentation in renal tubular cells in diabetic rats. Furthermore, FMN restored the mitochondrial dynamics-related proteins both *in vitro* and *in vivo*. These results suggested that FMN could regulate mitochondrial dynamics to improve mitochondrial function.

Finally, we further evaluated the effects of FMN on the Sirt1/PGC-1α pathway *in vitro* and *in vivo*. Sirt1/PGC-1α pathway has been reported to regulate mitochondrial dynamics (Cui et al., 2021). Drp1 expression could be reduced by activation or overexpression of Sirt1 (Ding et al., 2018; Li et al., 2019) and overexpression of PGC-1α (Guo et al., 2015). While silencing Sirt1 and PGC-1α expression led to abnormal mitochondrial dynamics (Ding et al., 2018). Studies have shown that PGC-1α binds to Drp1 promoter (Ding et al., 2018; Lei et al., 2022). In our study, FMN restored the expression of Sirt1 and PGC-1α both *in vitro* and *in vivo*. Therefore, it is likely that FMN exerts beneficial effects on regulating mitochondrial dynamics *via* the modulation of Sirt1/PGC-1α pathway.

Ferroptosis is a cell death process driven by cellular metabolism and iron-dependent lipid peroxidation. It was reported that ferroptosis was involved in renal tubular cell death in DN and there were increased expression levels of acyl-CoA synthetase long-chain family member 4 (ACSL4) and decreased expression levels of glutathione peroxidase 4 (GPX4) in DN mice (Wang et al., 2020). Previous study demonstrated that peroxiredoxin six overexpression eliminated high-glucose induced ferroptosis, which was reflected in the inhibition of iron accumulation and the increased expression of SLC7A11 and GPX4 (Zhang et al., 2021). Previous study revealed that the restraint of iron availability through blocking CD71-mediated iron endocytosis damaged the differentiation and pathogenicity of TH 17 cells (Li et al., 2021). We did not examine the changes of iron death-related factors such as SLC7A11, CD71, ACSL4 and GPX4. The effects of FMN on these iron death-related factors will be further examined in our further study.

According to the previous study (Oza and Kulkarni, 2019), FMN was administered orally to the diabetic animals at the dose of 10, 20 and 40 mg/kg. In our preliminary experiments, we found that 20 mg/kg of FMN ameliorated albuminuria and did not cause apparent toxicity to the kidney. Thus, we selected this optimal drug concentration in our study.

Moreover, we used the HE and PAS staining on the serial kidney sections and also measured the levels of ALT and Scr to evaluate the safety of FMN in rats. We found that FMN did not affect the levels of ALT and Scr. FMN had no obvious toxicity to the kidney as shown in HE and PAS staining. These findings suggested that FMN did not cause apparent toxicity to livers and kidneys.

However, there are some limitations in our study. Firstly, we did not assess the effects of FMN on the normal control group and these effects will be investigated in our further study. Secondly, we only investigate the beneficial effects of FMN on renal tubular cell injury and mitochondrial dysfunction in DN animals and the protective effects of FMN on podocyte injury needs to be studied in the further study.

Taken together, our study clearly demonstrated that FMN attenuated albuminuria, renal tubular injury and mitochondrial damage in diabetic rats partly through regulating Sirt1/PGC-1α pathway. These findings might provide a potential novel therapeutic strategy for DN.

## Data Availability

The raw data supporting the conclusion of this article will be made available by the authors, without undue reservation.
